# Are patients willing to accept longer travel times to decrease their risk associated with surgical procedures? A systematic review

**DOI:** 10.1186/s12889-020-8333-5

**Published:** 2020-02-19

**Authors:** Stefanie Bühn, Jakob Holstiege, Dawid Pieper

**Affiliations:** 1 Institute for Research in Operative Medicine, Faculty of Health, School of Medicine, University Witten/Herdecke, Ostmerheimer Str. 200, Building 38, D-51109 Cologne, Germany; 2 Central Research Institute of Ambulatory Health Care in Germany (Zi), Salzufer 8, D-10587 Berlin, Germany

## Abstract

**Background:**

Distance to a hospital is an influencing factor for patients´ decision making when choosing a hospital for surgery. It is unclear whether patients prefer to travel further to regional instead of local hospitals if the risk associated with elective surgery is lower in the farther hospital. The aim of our systematic review was to investigate patient preferences for the location of care, taking into consideration surgical outcomes and hospital distance.

**Methods:**

MEDLINE (PubMed), EconLit, PsycInfo and EMBASE were searched until November 2019. We included experimental choice studies in which participants were asked to make a hypothetical decision where to go for elective surgery when surgical risk and/or distance to the hospitals vary. There was no restriction on the type of intervention or study. Reviewers independently extracted data using a standardized form. The number and proportion of participants willing to accept additional risk to obtain surgery in the local hospital was recorded. We also extracted factors associated with the decision.

**Results:**

Five studies exploring participants´ preferences for local care were included. In all studies, there were participants who, independently of a decreased mortality risk or a higher survival benefit in the regional hospital, adhered to the local hospital. The majority of the patients were willing to travel longer to lower their surgical risk. Older age and fewer years of formal education were associated with a higher risk tolerance in the local hospital.

**Conclusions:**

Whether patients were willing to travel for a lower surgery-associated risk could not be answered in a straightforward manner. The studies we identified showed that decision making also relies on factors other than on rational information on risk or distance to hospital.

**Trial registration:**

International prospective register of ongoing systematic reviews (PROSPERO): CRD42016033655. Registered 1 January 2016.

## Background

Studies show that patients who make an active decision rarely rely only on a completely rational decision-making process but rather use other than rational comparative information. Patients´ active decision making is described as an active investigation and acquisition of information to make a conscious decision on health-related issues [1]. A scoping review shows that patients’ decision-making process is complex and heterogeneous [1]. Patients’ decisions often rely on their previous experiences in a hospital or on social influences such as recommendations by friends. Another influencing factor when choosing a hospital is accessibility. Patients prefer a health care provider that is nearby, so that travel time will be short. Younger age, higher education and willingness to travel in general are factors associated with more tolerance toward greater distances to a hospital. Some studies indicate that the distance to a hospital is even more important than medical outcomes [1–3]. Besides surgical reputation and surgical competency, hospital reputation and distance to the hospital are the primarily important attributes by which patients choose their surgeon [4]. In another study, previous personal experiences in the hospital were the most frequently stated criterion, followed by the hospital’s reputation, recommendation from one’s own outpatient caregivers, distance from home and recommendations from relatives [5].

In past years, numerous studies pointed out that in surgical disciplines in particular, there is a positive relationship between high-volume hospitals and outcomes, especially for mortality [6, 7]. To improve patients’ surgical outcomes, policy makers introduced minimum hospital volumes. Consequently, centralisation of these procedures occurs. Besides positive effects for the patients’ health on the one hand, centralisation of care to high-volume hospitals may lead to longer travel distances and increased travel burden. Patients might prefer local care for different reasons, for example, because it has advantages in terms of proximity to supportive family members and other local personal support systems, and it offers the possibility to receive a continuity of care [8, 9].

Distance to a hospital has a great impact on patients’ decision making, as shown by numerous studies [1–4, 10, 11]. Our main research aim is to investigate whether and to what extent patients consider surgical risk and travel distances in their decision-making process when choosing a hospital for elective surgery.

With our systematic review, we aim to identify choice experimental studies investigating patient preferences for the location of a hospital for elective surgery, taking into consideration surgery-associated outcomes and hospital distance. Our study should contribute to the discussion about the need to include patients’ preferences and values when making health care decisions about centralisation of clinical care and minimum surgery volumes.

## Methods

### Protocol and registration

This systematic review was registered with the international prospective register of systematic reviews (PROSPERO) (https://www.crd.york.ac.uk/PROSPERO/display_record.php?RecordID=33655).

### Eligibility criteria

We included experimental choice studies in which some kind of choice behavior measurement (trade-off, standard gamble, etc.) was performed. Participants needed to make a hypothetical decision on where to go for elective surgery when surgery-associated risk and/or distance to the hospitals (in terms of travel time or distance) vary. There was a restriction neither on the type of intervention nor on the medical discipline. There was no restriction on eligible underlying study types. Letters, editorials, and comments were excluded.

### Information sources

A systematic literature search in the MEDLINE (through PubMed), EconLit, PsycInfo and EMBASE bibliographic databases was performed from inception until November 2019 (DP, SB). References to the included studies were retrieved and assessed for relevance. Corresponding authors of the included studies were contacted to ask whether they knew any unpublished, recently published or ongoing studies that could be relevant to our review (SB). Our search strategy included search terms regarding distance and travel connected with patient preferences and choice experiments connected with search terms regarding risks and outcomes in the surgical field. The full electronic search strategy of MEDLINE is presented in Additional file 1. Search terms were adapted for every database searched.

### Study selection

Titles and abstracts were screened independently by two members (DP/JH or DP/ SB) of the research team. The full text of potentially eligible articles was retrieved, and two reviewers (DP, SB) independently assessed the eligibility of full texts against the review inclusion criteria. Any disagreement was resolved by discussion. When no agreement could be reached, a third member of the team was asked for final judgment. Data were extracted by one reviewer (SB) into piloted, structured summary tables and checked for accuracy and completeness by a second reviewer (JH). Any disagreement was resolved when consensus was reached.

### Data collection process

For each study, the main characteristics including country, the kind of scenario and methods to elicit preferences were extracted. Information about the setting, inclusion and exclusion criteria and travel time to the hospital were also extracted. As outcome, the number and proportion of participants willing to accept additional risk to obtain surgery in the local hospital was recorded. We also extracted factors associated with the decision.

### Risk of bias of individual studies

To judge the risk of bias (RoB) in the included studies, we referred to the Grading of Recommendations, Assessment, Development and Evaluation (GRADE) publication on assessing certainty of evidence in the importance of outcomes and patients’ preferences [12]. Domains in which the RoB should be assessed are: [1] selection of participants into the study, [2] completeness of data, [3] measurement instrument and [4] data analysis. We omitted the second domain (completeness of data) because in the identified studies, the no-response rate and the loss to follow-up were not involved. The quality of the included studies was rated by two reviewers (SB, DP) independently, and consensus was reached by discussion.

## Results

### Study selection

**Fig. 1:**
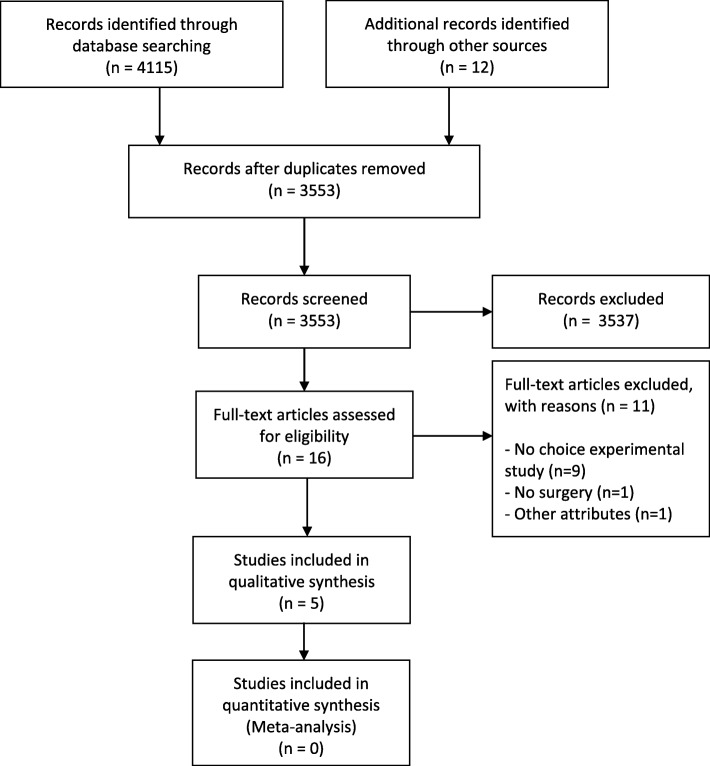
Flowchart

After screening 3553 titles and abstracts, 16 full-text publications were included for a detailed evaluation. Five studies met the inclusion criteria. The detailed study selection process is provided in Fig. 1. A list of the excluded studies with reasons is provided in Additional file 2.

### Study characteristics

We found three studies performed in the United States [8, 13, 14], one in Canada [9] and one in Germany [15]. All studies used hypothetical scenarios. In the study by Finlayson et al., the clinic’s inpatients and outpatients who were awaiting another kind of elective surgery were asked to imagine that they had potentially resectable pancreas cancer [8]. In the study by Landau et al., patients who were diagnosed with an asymptomatic abdominal aortic aneurysm (AAA), but had no planned surgery in the immediate future, were to imagine they had to undergo surgery for their AAA [9]. The third study, by Chang et al., included parents with children who were referred to hospital because of suspected heart abnormalities, but finally none were diagnosed which subsequently were not confirmed by diagnosis [13]. They were told to imagine that their children had to undergo open-heart surgery. Afterward, they were asked to imagine the situation for themselves. In the fourth study, patients who presented themselves for evaluation of a pelvic mass but with no proved cancer diagnosis, were asked to imagine that they were diagnosed with ovarian cancer and scheduled for surgery [14]. In the study by Burkamp et al., participants were asked to imagine that they had to make a decision on which hospital to choose for elective total knee arthroplasty [15].

### Characteristics of choice experiments of individual studies

To elicit patients’ preferences, all studies used discrete choice experiments (DCE). Patients were initially informed about the disease scenarios they should imagine for the DCE. The distances to local and regional hospitals and the surgery-associated risks were presented either as text, graphs, and pictograms or as a combination of these risk presentations. Three studies performed two different DCEs (see Table 1) [13–15]. Shalowitz et al. used different risk presentations with different distances (50 miles with an increased survival benefit in the regional hospital, and 0–250 miles in nine increments with a fixed risk presented). In the study of Chang et al., in which parents were requested to make a decision for their children and for themselves, they were presented with two different distances for the farther hospital. In all studies, DCEs started with an equal risk in both the local and regional hospital. Burkamp et al. used two different outcomes (risk of 90-day mortality and of revision). In total, four studies used mortality risk. In three studies, mortality risk in the local hospital increased stepwise [8, 9, 13] and in the study of Burkamp et al. [15], mortality risk and risk of revision in the regional hospital decreased. In the study of Shalowitz et al., the 5-year survival rate increased in the regional hospital.

**Table 1:** Characteristics of included studies

Study/ Country	Scenario/ Method	Setting/Inclusion/Exclusion	Travel time to hospital
**Finlayson** et al. (1999) Patient preferences for location of care; Medical Care, Vol. 37 (2) p.: 204–209 /USA	**Scenario:** hypothetical (participants should imagine that they had potentially resectable pancreas cancer) **Method:** discrete choice experiment (DCE)	**Setting:** hospital providing medical care to primarily male veterans from rural locations **Inclusion:** - consecutive sample of a clinic’s inpatients and outpatients who were awaiting elective surgery **Exclusion:** - inability to discuss operative risk due to impairment of mental capacity or high preoperative anxiety	Local: not reported Regional: 4-h drive
**Landau** et al. (2013) Determination of patient preferences for location of elective abdominal aortic aneurysm surgery; Vascular and endovascular surgery 47 (4) p.: 288–293/ Canada	**Scenario:** hypothetical (participants had abdominal aortic aneurysm [AAA] but no scheduled surgery in the immediate future) **Method:** discrete choice experiment (DCE)	**Setting:** university-affiliated medical centre providing vascular services for the surrounding population of 1.9 million people **Inclusion:** - patients with infra-renal, asymptomatic AAA (4–5 cm largest diameter) -living ≥1-h drive to hospital -patients not scheduled to undergo AAA surgery in the near future **Exclusion:** not reported	Local: 30 min’ drive Regional: 2-h drive
**Chang** et al. (2004) Parental preferences regarding hospitals for children surgery: A trade-off between travel distance and potential outcome improvement; Canadian Journal of Cardiology. 20 (9) p.:877–882/ USA	**Scenario:** hypothetical (children of participants referred to hospital because of heart murmur or chest pain but no heart abnormality was determined) Participants should imagine their children needed heart surgery. Afterward, parents should imagine having surgery and should make the decision for themselves) **Method:** discrete choice experiment (DCE)	**Setting:** paediatric cardiology clinic, primarily serving the indigent population (38% uninsured) **Inclusion:** - parents or adult primary caregivers of children referred to paediatric cardiology without upcoming surgery **Exclusion:** - determined cardiac abnormalities	Local: 10 min’ drive Regional: 1st scenario: 2-h drive 2nd scenario: 4-h drive
**Shalowitz** et al. (2018) Are patients willing to travel for better ovarian cancer care?; Gynecologic Oncology 148 (2018) p:42–48/ USA	**Scenario:** hypothetical (participants should imagine they have been diagnosed with ovarian cancer and initial cancer treatment [preoperative visit, hospitalisation for surgery, postoperative visit] should start) **Method:** discrete choice experiment (DCE)	**Setting:** one of two gynaecologic oncology clinics affiliated with a university **Inclusion:** - patients with suspected ovarian neoplasm **Exclusion:** - cancer diagnosis	DCE 1: Local: distance that participants travelled to reach the clinic Regional: additional 50 miles DCE 2: Distances between hospitals ranged from 0 miles (hospitals were equidistant from the participant’s home) to 250 miles in nine increments
**Burkamp** et al. (2019) Patient preferences between minimum volume thresholds and nationwide healthcare provision: the example of total knee arthroplasty; Z Orthop Unfall. 2019 / Germany	**Scenario:** hypothetical (participants should imagine that they had to undergo total knee arthroplasty) **Method:** discrete choice experiment (DCE)	**Setting:** recruited via random samples of registration offices and hospitals, DCE took place in hospital or in an office in the recruitment place **Inclusion:** - age 50–69 years - ability to understand the language and the DCE **Exclusion: not reported**	Local: 15 min’ drive Regional: 90 min’ drive

### Results of the DCEs of the individual studies

Participants’ preferences for local care varied between studies (see Table 2 and Additional file 3). In all studies, there were participants who, independently of a decreased mortality risk [8, 9, 13, 15], revision risk [15] or a higher survival benefit [14] in the regional hospital, adhered to the local hospital (3–10.7%).In the study by Finlayson et al., nearly half of the participants (45%) were willing to accept an additional risk to receive care locally [8]. They stratified by strength of preferences for local care, which showed that 21% of the participants accepted very high levels of additional risk (10 to > 50 percentage points) rather than going to a regional hospital. Ten percent of participants chose local care, even if the mortality rate was 100%.In contrast, Landau et al. found that the majority of participants (91%) did not tolerate any additional risk to have surgery performed locally [9].In all studies, a proportion of participants (1–40%) preferred the regional hospital for treatment although mortality rates were identical in both hospitals. Only in the studies by Finlayson et al. and Burkamp et al. (for the risk of revision), all participants (100%) preferred to go to the local hospital when risks were equal. The study by Chang et al. showed that parents were more willing to travel for their children’s care than for their own to keep mortality risk low, and that their decision depended on the travel distance. If the regional hospital distance was two driving-hours, participants more often chose the distant hospital compared to a 4-h driving distance.In Finlayson et al., 45% of the participants preferred surgery the regional hospital after the risk at the local hospital was increased in the first step. In the study by Chang et al., 36.9% (2 h-driving distances) respectively 48.5% (4-h drive) preferred to go to regional hospital for their child’s surgery when the first increase of the risk in the local hospital was performed. In the first DCE of Shalowitz et al., where the distance to regional hospital was 50 miles, 29% of the participants chose the local hospital for treatment of ovarian cancer when survival was slightly higher in regional hospital (34% versus 36%). Burkamp et al. performed two DCEs, one for 90-day mortality risk and one for risk of revision. For the risk of mortality, 92 of 180 participants (51%) chose the regional hospital for surgery when mortality risk decreased from 1 to 0.8% in regional hospital. When revision risk in regional hospital decreased by 2 percentage points (10 to 8%), 37% of the participants chose surgery in distant hospital.

**Table 2:** Results of choice experiments

Study (number of patients)	Outcome risk in local hospital	Outcome in regional hospital	Proportions of participants remaining for surgery in local hospital N (%)
**Outcome: mortality**			
Finlayson (*N* = 100)^a^	3%	3%	100 (100%)
	6%	3%	45 (45%)
	12%	3%	23 (23%)
	18%	3%	18 (18%)
	23%	3%	17 (17%)
	100%	3%	10 (10%)
**Outcome: mortality**			
Landau (*N* = 67)	2%	2%	40 (60% ^b^)
	3%	2%	6 (9%)
	4%	2%	5 (7.5%)
	9%	2%	2 (3%)
	32%	2%	1 (1.5%)
**Outcome: mortality**			
Chang† (*N* = 103)	3%	3%	85 (82.5%)
Scenario 1	4%	3%	38 (36.9%)
	6%	3%	20 (19.4%)
	18%	3%	10 (9.7%)
Scenario 2	3%	3%	85 (82.5%)
	4%	3%	50 (48.5%)
	6%	3%	23 (22.3%)
	18%	3%	11 (10.7%)
**Outcome: 5-year survival benefit**			
Shalowitz†† (*N* = 62)	34%	34%	42 (68%)
	34%	36%	18 (29%)
	34%	38%	13 (21%)
	34%	40%	12 (19%)
	34%	42%	7 (11%)
	34%	44%	6 (10%)
	34%	46%	2 (3%)
	34%	48%	2 (3%)
	34%	50%	2 (3%)
	34%	52%	2 (3%)
	34%	54%	2 (3%)
**Outcome: mortality**			
Burkamp (*N* = 180)	1%	1%	178 (99%)
	1%	0,8%	92 (51%)
	1%	0,6%	51 (28%)
	1%	0,4%	35 (19%)
	1%	0,2%	25 (14%)
	1%	0%	11 (6%)
**Outcome: revision**			
Burkamp (*N* = 180)	10%	10%	180 (100%)
	10%	8%	67 (37%)
	10%	6%	25 (14%)
	10%	4%	15 (8%)
	10%	2%	10 (6%)
	10%	0%	6 (3%)

^a^In original study, there were differences in presentation of the results in figures and tables; we refer to the results presented in the result section of the study

^b^Sixty-one participants who were not accepting any additional risk and number of participants accepting additional risk to keep surgery locally (number calculated)

† Only numbers of the decision of parents for their child’s surgery reported in the original study

††Only DCE1 displayed

### Associated factors

Shalowitz et al. compared patient characteristics of those who were willing to travel an additional 50 miles for a 6% survival benefit and those who were not willing. Participants who were not employed (56% vs. 83%, *p* = 0.05) and who rated their own health as good to excellent (86% vs. 50%, *p* = 0.04) were more likely to travel. Landau et al. identified characteristics of patients who preferred regional care if risks in both hospitals were equal. A previous treatment in the regional hospital and presence of others living in the home were factors associated with acceptance to travel longer distances.

Finlayson et al. performed an ordinal logistic regression analysis and identified older age and fewer years of formal education with a higher acceptance of additional risk of surgery in a local hospital. Burkamp et al. performed a multivariate regression analysis to identify factors associated with patients’ decisions. Surgery in the local hospital was preferred despite higher mortality risk by study participants who were recruited by registration office (in contrast to hospital recruitment), had a lower educational level, subjectively attached greater importance to hospital distance, or had friends or acquaintances with prior knee replacement surgery in that hospital. With regard to risk of revision, accessibility by public traffic and satisfaction with the number of visitors during a 10-day hospital stay were associated with preference for local care [15].

### Risk of bias of included studies

Critical appraisal of the studies showed that three out of five studies [8, 9, 13] had a serious risk of bias (RoB). The RoB for the studies by Shalowitz et al. and Burkamp et al. was rated as moderate [14, 15]. In domain one, the selection of participants into the study, two studies showed a critical RoB [8, 13], one study had a serious [9] and another two a moderate RoB [14, 15]. In the second domain, which asked whether the instrument used for eliciting the relative importance of outcomes was valid and administered in an appropriate way, all but two studies [14, 15] showed a serious RoB. In the third domain (data analysis), the RoB was rated moderate in all studies. An overview of the RoB for the included studies is provided in Table 3.

**Table 3:** Risk of bias

	Finlayson	Landau	Chang	Shalowitz	Burkamp
**Domain 1: Study population**					
*1. Was an appropriate study sample selected from the sampling frame?*	N	PN	N	PY	PY
Risk of bias in domain 1	critical	serious	critical	moderate	moderate
**Domain 2: Instrument**					
*2. Was the chosen instrument for eliciting relative importance of outcomes valid and reliable?*	PN	PN	PN	PN	PN
*3. Was the instrument administered in the intended way?*	PY	PY	PY	PY	PY
*4. Was a valid representation of the outcome*^*a*^ *used?*	PN	PN	PN	Y	Y
*5. Did the researchers check the understanding of the measurement instrument?*	The investigators did not formally test the understanding, but there was evidence suggesting inadequate understanding.	The investigators did not formally test the understanding, but there was evidence suggesting inadequate understanding.	The investigators did not formally test the understanding, but there was evidence suggesting inadequate understanding.	The investigators did not formally test the understanding, but there was evidence suggesting adequate understanding.	The investigator tested the understanding and understanding was adequate
Risk of bias in domain 2	serious	serious	serious	moderate	moderate
**Domain 3: Analysis**					
*6. Were the results analysed appropriately to avoid influence of bias and confounding?*	PY	PY	PY	PY	PY
Risk of bias in domain 3	moderate	moderate	moderate	moderate	moderate
**Risk of bias within study**	serious	serious	serious	moderate	moderate

Modification of Zhang, Y., et al., *GRADE Guidelines: 19. Assessing the certainty of evidence in the importance of outcomes or values and preferences -- Risk of bias and indirectness.* J Clin Epidemiol, 2018 [10].

^a^RoB rated for outcomes mortality risk, revision and survival; *N* No, *PN* probably no, *PY* probably yes, *Y* Yes

## Discussion

Our systematic review identified five studies that examined patients’ preferences when trading off between surgery-associated risks and hospital distances. Both the surgery-associated risk and the distance seemed to have an influence on patients’ decision making. In all studies, there was a general trend toward acceptance of greater travel distances if the surgical risk in the local hospital increased. However, in all studies, there was a fraction of participants who, despite a maximally increased risk, still preferred to undergo surgery in the local hospital (see Table 2). This leads to the assumption that, besides risk and distance, there might be other factors that have an influence on patients’ decision-making process. One study identified unemployment and a rating of one’s own health as good to excellent as characteristics associated with greater acceptance to travel [14]. Two studies performed regression analyses to identify participants’ characteristics associated with the decision for the local hospital even if mortality risk there was higher [8, 15]. Older age and fewer years of formal education were associated with preferences for local care in the study of Finlayson et al. [8]. Burkamp et al. also identified lower school-leaving qualifications as a factor associated with the willingness to accept higher mortality risks in the local hospital [15].

### Heterogeneity between the studies identified

There was great variation in the characteristics of the included studies. The diseases used in the DCEs differed regarding severity and prognosis, which might have influenced choice behavior and willingness to travel. First, prognosis of diseases used in the scenarios varied greatly. One study used pancreatic cancer, which is one of the deadliest cancer types, with a 5-year relative survival rate of 6% for men and 8% for women in Europe [16]. Another study chose open-heart surgery (i.e., ventricular septal defect), which in contrast has a good prognosis [17].Second, despite the generally hypothetical nature of the DCEs there were differences between studies regarding the degree patients were affected with the scenario which likely influenced their ability to imagine being in the hypothetical situation. There were also differences regarding the study population. Although all studies used a hypothetical scenario, in one study [9], participants really suffered from the disease, which might have had an impact on the decision-making process. In one study, participants had the clinical suspicion of ovarian cancer and were referred to the clinic for clarification [14]. In contrast, in the study by Chang et al. [13], parents already knew that their children did not have heart abnormalities [13]. Therefore, decision making in the first study [14], where participants might receive a cancer diagnosis, might have been influenced by anxiety. In one study, patients had to make a decision between a local hospital and the regional hospital in which the study was conducted [9]. Third, in another study, participants were asked to imagine the local hospital was the hospital where the family in general receives care [13]. Research showed that a previously made personal experience of patients in a hospital was an influencing factor [5].

### Differences in risk presentation

The kind of risk presented in the choice experiments differed between studies. One study used 5-year survival benefit [14], whereas the other studies used mortality risk [8, 9, 13]. Studies show that there are differences in understanding risks and that the framing of the risk attribute (positive [survival] versus negative [mortality]) has an influence on decision-making behaviour in a DCE [18]. Negative risk framing leads to more risk-seeking behaviour. Presentation of the risks of the choice experiments also differed, and only Shalowitz et al. and Burkamp et al. referred to evidence-based risk communication such as presentation of risks with both words and pictograms. Adequate understanding of the risks presented is an essential precondition to eliciting participants’ preferences for local care, so it remains questionable whether the data presented in the studies reflects patients´ actual preferences. Especially in Finlayson et al., when participants would stay in the local hospital even if they had a 100% mortality risk, the question arises whether they had properly understood the risks and consequences presented. In the majority of patients, health literacy is low. In addition, risk presentation was found to often be ambiguous [19]..

### Results of other studies

Previous studies have identified additional factors influencing patients’ choice of hospital for treatment.. The scoping review of Victoor et al. found that patients consider a variety of structural (e.g., availability), process (e.g., waiting time) and outcome (e.g., mortality rate) characteristics of providers [1]. Other studies in this field identified hospital and surgical reputation, surgical competency, personal experiences the patient had in a hospital and recommendations from relatives or outpatient personnel as influencing factors [4, 5]. A study by Varkevisser et al. with empirical data on revealed preferences of patients with orthopaedic and neurosurgical surgery showed that extra travel time and good hospital waiting time performance affected the decisions to visit the hospital closest to patients’ homes [11].

### Strengths and limitations

To the best of our knowledge, this is the first systematic review on patients’ preferences evaluating the trade-offs between distance to hospital and surgery-associated risks using choice experiments. Our study has several limitations. First, although we performed a systematic search and contacted study authors, we might have missed some relevant studies. The second limitation is that the included studies had a moderate to high risk of bias. To assess the RoB of the patients’ preference studies we used a recently developed tool [12]. However, to the best of our knowledge it is not yet validated. Another limitation is that generalisability of the study findings is limited. All studies used a hypothetical scenario in which patients were asked to make a decision based on an imagined situation. The impact of the differences in choice behaviour between participants who were really suffering the disease and those who were not remains unclear. However, research shows that discrete choice experiments (DCEs) could predict real-world decisions [20]. Results of the studies might not be transferable to other health care systems and settings. In countries with relatively large geographical distances and low population density, such as Australia, the definition of reasonable distance to a hospital might differ compared to countries with higher population density [21]. Two of the authors (SB, DP) co-authored the study of Burkamp et al. [15]. Although we were aware of this conflict of interest, we could not completely exclude an influence on the quality rating of this study [22].

## Conclusions

In all studies, there was a general trend toward the acceptance of greater travel distances if the surgical risk in the local hospital increased. The question of whether patients were willing to travel for a lower surgery-associated risk could not be answered in a straightforward manner. The studies we identified showed that decision making does not only rely on rational information such as risk information and distance to hospital. Against the background of centralisation of clinical care and minimum surgery volumes, patient preferences regarding hospital choice are crucial to be considered by national policy decision makers. Our study shows a high need for more studies that consider patients’ preferences and values. Future studies should investigate the influence of hospital distance on decision making, comparing diseases with different severity and prognosis, comparing specialised surgery with routine procedures. Risk presentation should rely on the newest risk communication research and a proper understanding that the choice experiments should be validated by pretesting the scenarios. Participants living in different settings (urban, suburban and rural) should be included to investigate the influence on willingness to travel.

## Supplementary information

**Additional file 1.** Search strategy Medline

**Additional file 2.** List of excluded studies

**Additional file 3.** Bar graphs

## Data Availability

The datasets used and/or analyzed during the current study are available from the corresponding author on reasonable request.

## Abbreviations

AAA: Abdominal aortic aneurysm
DCE: Discrete choice experiment
GRADE: GRADING of Recommendations, Assessment, Development and Evaluation
PROSPERO: Prospective register of ongoing systematic reviews
RoB: Risk of Bias
