# Supplementary material for: Are patients willing to accept longer travel times to decrease their risk associated with surgical procedures? A systematic review
Source: BMC Public Health. 2020 Feb 19;20:253. doi: 10.1186/s12889-020-8333-5 (PMC7031936; doi:10.1186/s12889-020-8333-5)
Supplement: Supplementary file 1 — Additional file 1. Search strategy Medline [file 12889_2020_8333_MOESM1_ESM.doc]

**Appendix 1**

Search strategy Medline (via Pubmed)

(Travel*[tiab] OR "Travel"[Mesh] OR distance[tiab] OR regional*[tiab] OR local*[tiab] OR rural[tiab] OR “Catchment Area (Health)"[Mesh])
AND
(Prefer*[tiab] OR "Patient Preference"[Mesh] OR "Consumer Behavior"[Mesh] OR "Decision Making"[Mesh] OR decide[tiab] OR decision*[tiab] OR “standard gamble”[tiab] OR “trade off”[tiab] OR “choice experiment”[tiab] OR “choice behavior” [tiab] OR “choice behaviour” [tiab])
AND
(Risk*[tiab] OR "Risk"[Mesh] OR probability*[tiab] OR "Probability"[Mesh] OR outcome*[tiab] OR rate*[tiab] OR "Outcome Assessment (Health Care)"[Mesh])
AND
(Hospital*[tiab] OR clinic[tiab] OR clinics[tiab] OR "Hospitals"[Mesh] OR facility[tiab] OR facilities[tiab] OR centre [tiab] OR center [tiab])
AND
(Surgery[tiab] OR surgical[tiab] OR "Specialties, Surgical"[Mesh] OR operative[tiab] OR elicit*[tiab])
