# Supplementary material for: Are patients willing to accept longer travel times to decrease their risk associated with surgical procedures? A systematic review
Source: BMC Public Health. 2020 Feb 19;20:253. doi: 10.1186/s12889-020-8333-5 (PMC7031936; doi:10.1186/s12889-020-8333-5)
Supplement: Supplementary file 2 — Additional file 2. List of excluded studies [file 12889_2020_8333_MOESM2_ESM.doc]

| **Excluded studies** | **Reasons** |
| --- | --- |
| Damman, O.C., et al., Creating compact comparative health care information: what are the key quality attributes to present for cataract and total hip or knee replacement surgery? Med Decis Making, 2012. 32(2): p. 287-300. | No choice experimental study |
| Fitch, M.I., et al., Travelling for radiation cancer treatment: Patient perspectives. Psycho-Oncology, 2003. 12(7): p. 664-674. | No choice experimental study |
| Fitzgerald, J.D., et al., Potential impact on patient residence to hospital travel distance and access to care under a policy of preferential referral to high-volume knee replacement hospitals. Arthritis Care and Research, 2012. 64(6): p. 890-897. | No choice experimental study |
| Jan, J., et al., The use of conjoint analysis to elicit community preferences in public health research: A case study of hospital services in South Australia. Australian and New Zealand Journal of Public Health, 2000. 24(1): p. 64-70. | Other attributes |
| Kløjgaard, M.E., et al., Patient preferences for treatment of low back pain - A discrete choice experiment. Value in Health, 2014. 17(4): p. 390-396. | No surgery |
| Luft, H.S., et al., Does quality influence choice of hospital? JAMA, 1990. 263(21): p. 2899-906. | No choice experimental study |
| Patiar, S., et al., Patient satisfaction with NHS elective tonsillectomy outsourced to the private sector under the Patient Choice Programme. J Eval Clin Pract, 2006. 12(5): p. 569-72. | No choice experimental study |
| Shackley, P., R. Slack, and J. Michaels, Vascular patients' preferences for local treatment: An application of conjoint analysis. Journal of Health Services Research and Policy, 2001. 6(3): p. 151-157. | No choice experimental study |
| Varkevisser, M. and S.A. Van Der Geest, Why do patients bypass the nearest hospital? An empirical analysis for orthopaedic care and neurosurgery in the Netherlands. European Journal of Health Economics, 2007. 8(3): p. 287-295. | No choice experimental study |
| Weeks, W.B., et al., Veterans' care preference for coronary artery bypass grafting in a rural setting. Military Medicine, 2002. 167(7): p. 556-559. | No choice experimental study |
| Yahanda, A.T., et al., A Systematic Review of the Factors that Patients Use to Choose their Surgeon. World J Surg, 2016. 40(1): p. 45-55. | No choice experimental study |

Appendix 2: List of excluded studies
