# Supplementary figures and images for: Are patients willing to accept longer travel times to decrease their risk associated with surgical procedures? A systematic review
Source: BMC Public Health. 2020 Feb 19;20:253. doi: 10.1186/s12889-020-8333-5 (PMC7031936; doi:10.1186/s12889-020-8333-5)

Appendix 3

Bar graphs - Proportion of participants choosing the local hospital for surgery (%)


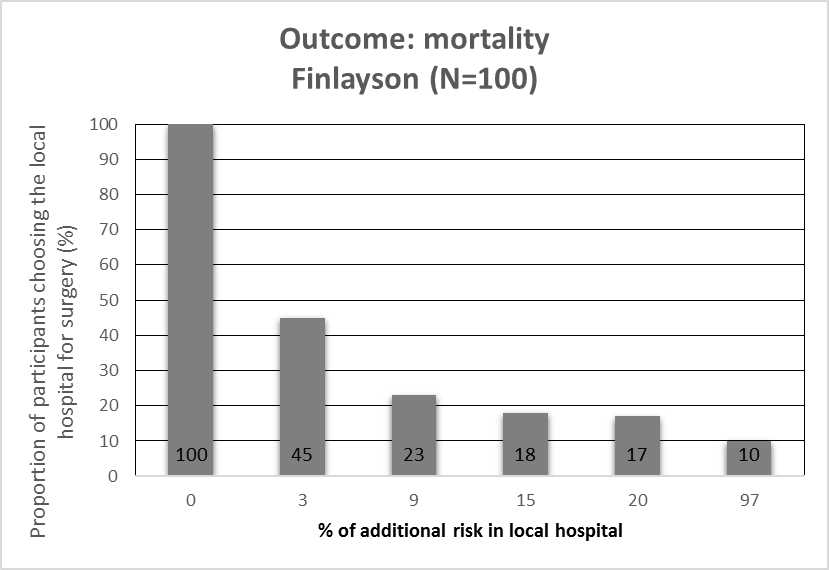


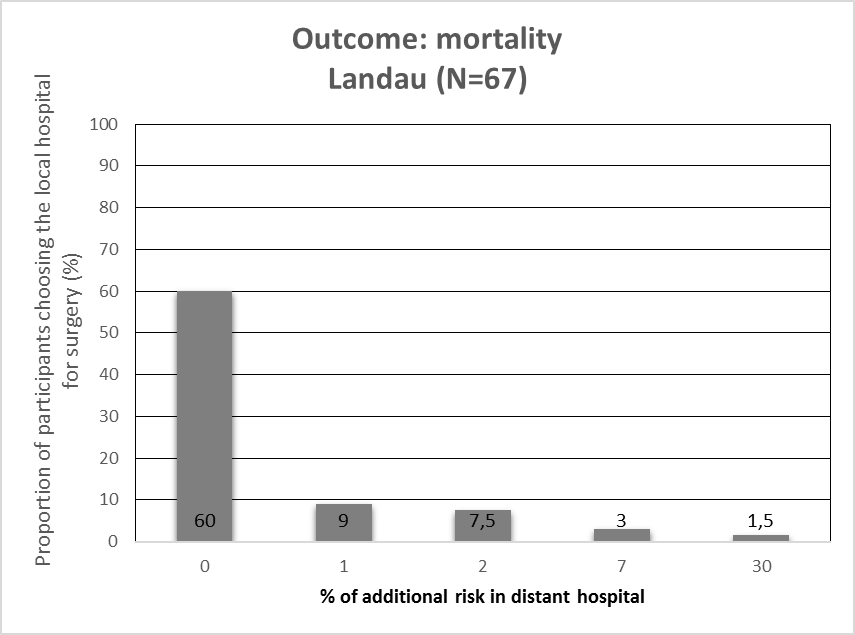


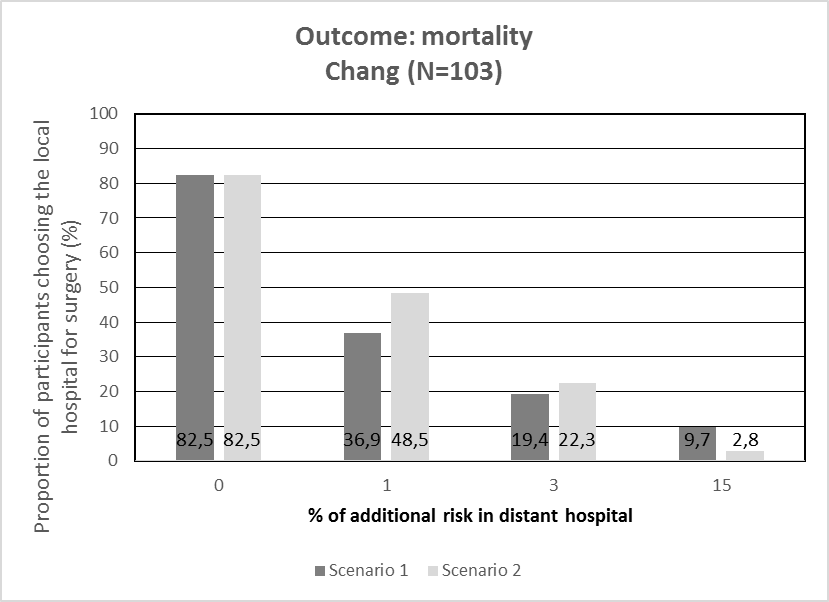


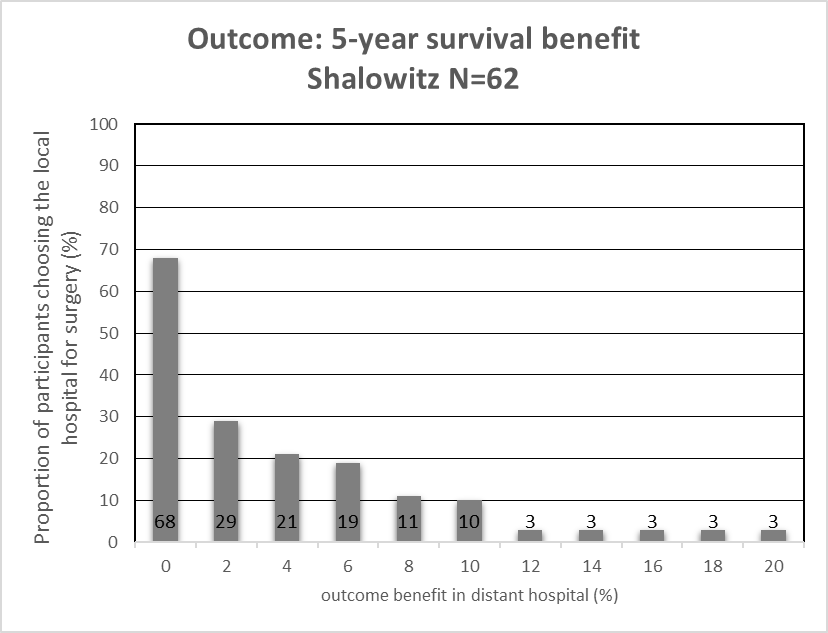


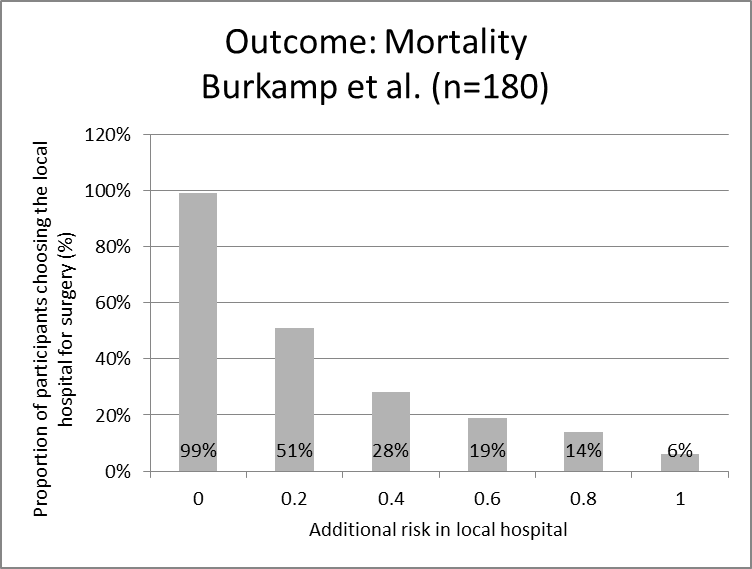


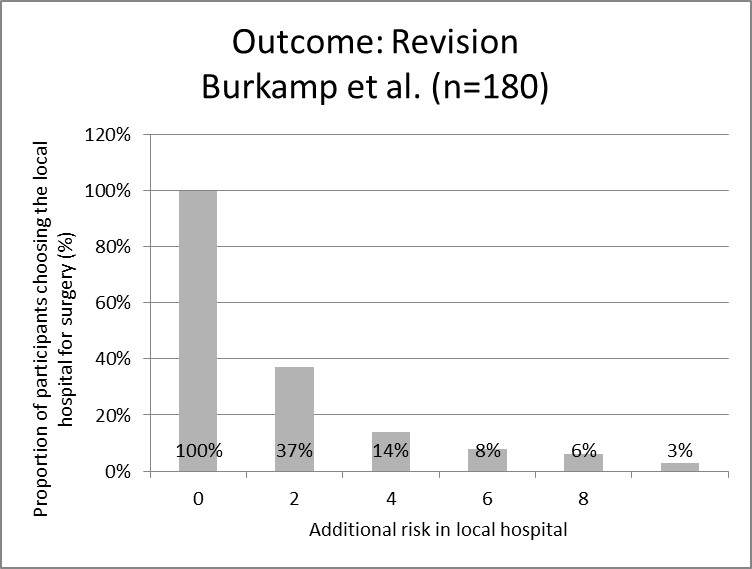

Supplement: Supplementary file 3 — Additional file 3. Bar graphs [file 12889_2020_8333_MOESM3_ESM.doc]
